# The Mobile and Pinned Grain Boundaries in 2D Monoclinic Rhenium Disulfide

**DOI:** 10.1002/advs.202001742

**Published:** 2020-10-12

**Authors:** Fangyuan Zheng, Lingli Huang, Lok‐Wing Wong, Jin Han, Yuan Cai, Ning Wang, Qingming Deng, Thuc Hue Ly, Jiong Zhao

**Affiliations:** ^1^ Department of Applied Physics The Hong Kong Polytechnic University Kowloon Hong Kong China; ^2^ Department of Chemistry and Center of Super‐Diamond & Advanced Films (COSDAF) City University of Hong Kong Kowloon Hong Kong China; ^3^ City University of Hong Kong Shenzhen Research Institute Shenzhen 518057 China; ^4^ Physics Department and Jiangsu Key Laboratory for Chemistry of Low‐Dimensional Materials Huaiyin Normal University Huaian 223300 China; ^5^ Department of Physics Hong Kong University of Science and Technology Clear Water Bay Hong Kong China

**Keywords:** 2D materials, density functional theory, grain boundaries, kinetics, transition electron microscopy

## Abstract

In bulk crystals, the kinetics of dislocations is usually hindered by the twining boundaries (TB) or grain boundaries (GB), rendering the well‐known grain boundary strengthening effects. Nevertheless, here it is found that in 2D rhenium disulfide (ReS_2_), twinning is much easier than dislocation slip. Consequently, the highly mobile TBs or GBs are inversely pinned by the relatively immobile dislocations. Due to the strong in‐plane covalent bonding, the GBs in high‐symmetry 2D materials such as graphene which consists of defects are immobile at room temperature. In contrast, in monoclinic 2D ReS_2_ several types of GBs (including TBs) can be readily generated and driven by mechanical loading. A complete library of the GBs in 2D ReS_2_ is established by the (in situ) atomic‐scale transmission electron microscopy (TEM) characterizations and density functional theory (DFT) calculations. The twinning (shear) stresses for 2D ReS_2_ are estimated as low as 4–30 MPa, one or two orders of magnitude lower than the traditional bulk materials. Full elucidation on the GB structures and especially the intriguing GB kinetics in such anisotropic 2D materials are of fundamental importance to understand the structure–property relationships and develop strain‐tunable applications for 2D materials in future.

## Graphical abstract

A complete library of grain boundaries in 2D rhenium disulfide, classified by the mobile and pinned nature, is established. In 2D rhenium disulfide twinning is much easier than dislocation slip. As a result, the highly mobile twining boundaries (TBs) or grain boundaries (GBs) can be inversely pinned by the relatively immobile dislocations.

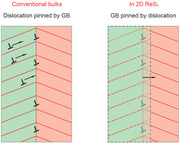

## 1 Introduction

The structures and novel properties of emergent 2D materials have been unveiled over the last decade.^[^ ^1^ ^]^ From the high‐symmetry graphene,^[^ ^2^, ^3^ ^]^ to the low‐symmetry black phosphorous (BP),^[^ ^4^, ^5^ ^]^ numerous 2D van der Waals (vdW) layered materials with monoatomic thickness have been realized.^[^ ^6^, ^7^, ^8^, ^9^ ^]^ Very recently, monoclinic 2D materials such as rhenium disulfide (ReS_2_) and rhenium diselenide (ReSe_2_) with the ever lowest symmetry in 2D have been fabricated and drawn much attentions for their outstanding anisotropic properties.^[^ ^10^, ^11^ ^]^ On the other hand, it has been shown that the grain boundaries (GBs) prevalently existed in many 2D materials especially synthesized by chemical vapor deposition (CVD) growth.^[^ ^12^ ^]^ The remarkable electrical^[^ ^13^ ^]^ and mechanical effects^[^ ^14^ ^]^ from these GBs on 2D materials have been highlighted. Regarding the atomic structures, the GBs in high‐symmetry 2D materials, such as graphene and molybdenum disulfide (MoS_2_) have been well documented.^[^ ^2^, ^15^ ^]^ The GBs in these high symmetry 2D materials consist of in‐plane dislocations or atomic defects; therefore, a relatively high energy barrier are requested to trigger the GB movement.^[^ ^16^, ^17^ ^]^ Herein, through the high resolution atomic‐scale transmission electron microscopy (TEM) characterizations and atomistic density functional theory (DFT) simulations, the GBs in 2D materials with lowest symmetry—the monoclinic 2D ReS_2_, have been fully understood. Apparently, the GB types are greatly enriched by the low‐symmetry structure, in particular the twinning boundaries (TB). Meanwhile, the distinct GB kinetics is also unusual and endows larger mechanical deformability to 2D ReS_2_.

The GBs in graphene can be classified into low‐angle (LA) twinning GBs made of piled‐up (Stone–Wales) dislocations and high angle (HA) irregular GBs.^[^ ^18^ ^]^ In 2D monolayer (1L) MoS_2_ or other similar hexagonal phase transition metal dichalcogenides (TMD), except for the LA twin GBs and HA GBs above, the break of inversion symmetry additionally introduces the 180° mirror GBs (or inversion GB).^[^ ^19^, ^20^ ^]^ Moreover, all the GBs in 2D MoS_2_ can be subdivided into two categories (“up” and “down”) due to lack of inversion symmetry. Different dislocation core structures belong to two respective categories have been observed by us.^[^ ^21^ ^]^ Generally, all these GBs observed in 2D materials are formed during the sample growth stages (viz. via thermodynamics), specifically, by the stitching of single‐crystalline flakes grown from different nuclei.^[^ ^22^ ^]^ Apart from growth, defect creation by electron irradiation and annealing could also generate new GBs within the pristine single‐crystalline graphene.^[^ ^23^, ^24^ ^]^ However, the generation, movement and annihilation of dislocations usually require high energies in most of the defect‐less 2D materials. Besides, owning to the high energy of in‐plane stacking fault (SF), the mechanical twinning process^[^ ^25^ ^]^ is normally not allowed in high‐symmetry 2D materials. As a result, the presence of GBs and lack of dislocation/twinning dynamics in 2D materials lead to poor ductility in polycrystalline 2D materials.^[^ ^26^ ^]^

## 2 Results and Discussions

**Figure 1:**
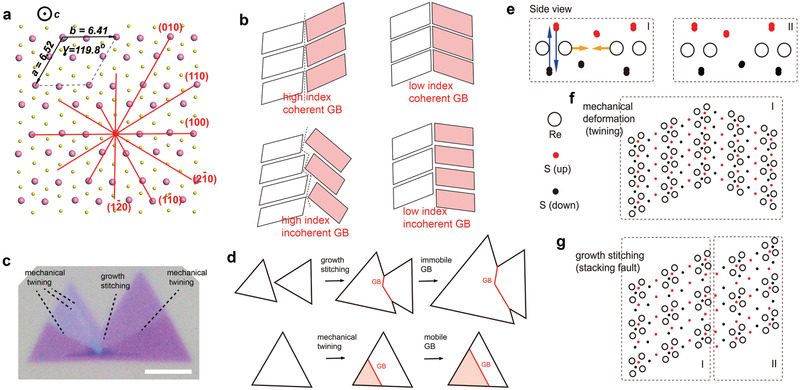
The mobile and pinned GBs in 1L ReS_2_. a) Schematic diagram of monoclinic atomic structure of 2D ReS_2_, six low‐index planes are highlighted. b) Schematic diagram of four groups of GBs in ReS_2_, divided by miller index and coherence at GBs. c) Polarized optical microscopy (OM) image showing grains and GBs in 1L‐ReS_2_ samples from CVD growth, the scale bar is 5µm. d) Two GB formation mechanisms. e) Side view arrangements of two mirrored S arrangement (I and II) in 1L‐ReS_2_, S atoms (upper (red) and lower (black) S atoms with respective to Re atomic plane) and Re atoms (black hollow circles) are shown. Blue arrows indicate the S atom movement during transition between I and II. Yellow arrows indicate the easy Re atom movement when the S lattice (either I or II) does not switch. f) Plane‐view of mobile boundaries (by mechanical loading) in 1L ReS_2_, consisting a single type S atom arrangement (either I or II). g) The stacking fault (SF) GB consisting of two grains with different (I and II) S arrangements, pinned by S lattices.

In contrast, 2D ReS_2_ has monoclinic atomic structure (**Figure** **1**a). The six S atoms are bonded with the central Re atom in distorted tetragonal (T′) coordination,^[^ ^27^ ^]^ leading to its unique domain architects, fundamentals, and applications.^[^ ^28^, ^29^, ^30^ ^]^ However, even some previous work studied the GBs in ReS_2_,^[^ ^31^ ^]^ the comprehensive understandings of mechanical kinetics and a clear classification of these GBs are still lacking. In line with the enhanced atomic dynamics on surfaces, the facile atomic reconstructions (including the Re diamond‐chain zipping/unzipping) in 2D ReS_2_ have been observed.^[^ ^32^ ^]^ In this connection, the twinning and TBs can be rendered by the lattice orientation switching in 2D ReS_2_. Basically, like the standard mechanical TBs, the TBs here can be nucleated and driven by mechanical straining in 2D ReS_2_. Here we classify all the GBs in ReS_2_ into four subgroups, by high/low‐index and coherent/incoherent, respectively (Figure 1b). The low/high‐index refers to the Miller Index for the GB planes (also used for the name of GBs below), see Figure 1a for the six low‐index planes in ReS_2_. High‐index usually refers to the vicinal planes of the low‐index planes. Coherency of GB is determined by the periods of the both side crystals at the GB planes, while coherent GB means a perfect matching and no interfacial strain.

Meanwhile, the GBs present in 2D 1L‐ReS_2_ samples grown by CVD (see Experimental Section) can be originated from two different mechanisms, viz., by direct growth and mechanical strain (Figure 1c,d). The flake stitching is quite similar to other 2D materials, but the deformation twining in 2D ReS_2_ is unique. It should also be noted that the lattices for sulfur (S) atoms may have two types, named as type I and II, which are in mirror symmetry (Figure 1e). Mechanical loading can reconstruct the lattices of Re atoms (following blue arrow direction) without transformation of the S lattices (following yellow arrow direction) between type I and II, explainable by the fact that rearrangement of S atoms needs to cross the basal plane, while Re atomic rearrangement not (Figure 1e). Thus, the mechanical twinning (Figure 1f) should possess pure I or II type S lattice in both side crystals, while the growth stitching (Figure 1g), especially the SF GB as we will introduce below, possess the mixed I/II type S lattices, in another word, they can be pinned by the S lattices.

**Figure 2:**
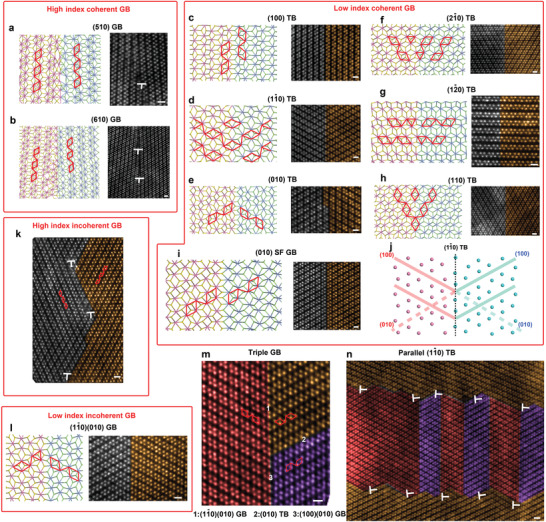
A Library of GBs in 1L ReS_2_: DFT calculated atomic models (left) and STEM‐ADF images (right). a,b) High index coherent GBs:(510), (610) lattice plane at GBs. Two side crystals of GBs are colored differently. Re diamond chains and dislocations are highlighted by red and white signs. c–h) Low‐index coherent Twinning Boundary (TB), corresponding to six low‐index planes in Figure 1a. i) Stacking fault (SF) GB, belonging to low‐index coherent GB. j) Scheme for lattice distortion crossing TBs. k) Nonstraight high‐index incoherent GB. l) One example for low‐index incoherent GB. m) A triple GB with three GBs crossing together. Specific GB types are indicated below by numbers. n) STEM figure parallel TBs encapsulated by piled up dislocations. Scale bar of STEM figures is 0.5 nm in all figures.

In our experiments, the as‐grown 1L ReS_2_ were transferred onto TEM grid by poly(methyl methacrylate) (PMMA) method,^[^ ^33^ ^]^ and then characterized by the probe aberration‐corrected scanning TEM (STEM) at the atomic scale (see Experimental Section). However, GBs from direct growth form with the gaseous kinetic process and multiple chemical phases, which increase the complexity to control them.^[^ ^33^ ^]^ It is suggested the nucleation sites and growing rate can be controlled by the flow rate of the vapor sources and seeds density (in Au‐quantum‐dots‐assisted vapor‐phase growth).^[^ ^34^ ^]^ A high flow rate allows more monolayer TMDs with decent sizes of flakes.^[^ ^35^ ^]^ During transfer, wrinkles, cracks, and other defects can be created in the sample (Figure S1, Supporting Information). For taking the GB images, the free‐standing membranes without such defects were chosen, and in the relatively flat area close to the edge of the flake where contains less strain. All the ReS_2_ GB atomic structures have been simulated by DFT method and directly matched with the experimental STEM‐annular dark field (ADF) images. First, we take a look at the coherent GBs (mainly twin GBs) with the least mismatch and internal strain. The coherent twin GBs can be sub‐divided into high‐index coherent GB (misorientation angle 0^°^—30°) (**Figure** **2**a,b) (see Figure S2 (Supporting Information) for the raw images) and low‐index coherent GB (Figure 2c–i). The former is usually constructed by 2D flake stitching during growth, and they consist of periodic dislocations, resembling ordinary LA GBs. In contrast, the latter (Figure 2c–h, except for the SF GB) is able to be formed by strain induced lattice reconstruction (twinning). The slightly distorted tetragonal structure in 2D ReS_2_ renders six types of low‐index TBs. Upon relaxation, the corresponding lattice shear angles for the twinning grains with respective to the original grains range from 1–3^°^, Figure 2j shows the ultrasmall shear angle (2.5°) for lattices (the red (100) plane and dashed blue (010) plane) over the two sides of (1–10) TB. Hence low mechanical stress is anticipated to enable the twining. If the orientation of TB is in between the orientations of the above six types (i.e., following higher index planes), the TBs can be decomposed into short segments of low‐index TBs possibly separated by dislocations (Figure S3, Supporting Information).

Interestingly, the twinning (lattice reconstruction) process in 2D ReS_2_ involves a synergistic bonding rearrangement between Re and S atoms. If we focus on the S atom positions, actually three types of TBs (Figure 2f–h) are exact twinning GBs for both Re and S lattice positions, while the other three types (Figure 2c–e) are TBs for only Re lattice positions, and inverse twin GBs for S lattice positions (Figure 1f). It is originated by the lack of inverse symmetry (with respect to *c* axis) in 1L ReS_2_. As we said, the S lattices are less likely to change the symmetry parity (switch between I and II types) under mechanical loading. This gives rise to a kind of special SF GB in 1L ReS_2_ (Figure 1g, Figure 2i). Both grains beside such GBs have the same orientation in terms of Re lattices, nevertheless, the S lattices not. Therefore, the stitching of such edges during growth is prone to produce a SF GB. If both the Re and S lattices are exactly same oriented, perfect stitching with no GBs will occur. In this instance, such SF GBs are not originated by mechanical loading. STEM image simulation shows the consistent results with all the STEM‐ADF images (Figure S4, Supporting Information).

Compared to the coherent GBs in 2D ReS_2_, incoherent GBs have much higher misfit strain at the GBs. The typical incoherent high angle (HA) (30°—180^°^) GBs (namely incoherent high‐index GB) in 2D ReS_2_ are shown in Figure 2k. The incoherent high‐index GBs can be considered as the combination of coherent high‐index GBs and incoherent (or coherent) low‐index GBs. In addition to the dislocations as seen in the coherent high‐index GBs, the greater lattice mismatches can bend the original straight GBs, decomposing them into zigzag low‐index GB segments separated by dislocations, and they are usually generated by growth stitching similar to the coherent high‐index GBs. Although the formation energies of the incoherent high‐index GBs are higher than coherent high‐index GBs, due to the inevitable high angle stitching during growth occasionally, these incoherent GBs can be widely found in CVD‐grown samples.

Conversely, the incoherent low‐index GBs (Figure 2l) are usually observed in bended GBs or complex strain zones, e.g., the triple GB cross (Figure 2m). The interfacial lattice misfit strains on the incoherent low‐index GBs are hence partially compensated by the circumferential strain relaxation factors, lowering the energy cost for the incoherent GBs. The incoherent low‐index GBs can also be pinned at defects, but in general, because the lattice mismatch at these incoherent low‐index GBs are quite low (1–2%) and the formation energies are relatively low, they are mobile and can freely interact with the aforementioned coherent TBs.

Due to the intrinsic lattice shearing at TBs and lattice mismatch at the incoherent low‐index GBs, they inherently tend to be pinned by the dislocations, more precisely, by the strain field of dislocations. One example can be found in Figure 2n, the parallel TBs are distributed between perpendicular LA GBs, nucleated and located at the dislocation cores. Overall, the GBs in monoclinic 2D ReS_2_ are summarized in **Table** **1**. The DFT calculations show the predominantly lower formation energies for coherent GBs, particularly the low‐index TBs. The SF GB has significantly higher energy because of S lattice mismatch.

**Table 1:** Summary and classification of all GBs in 1L ReS_2_

Classifications	Name	Formation energy (DFT) [eV nm^−1^]	Origin	Kinetics
Coherent high‐index GB		Medium	Growth	Pinned by dislocations
Coherent Low‐index GB	(100) TB	0.20	Mechanical twinning	Mobile
	(1–10) TB	1.16		
	(010) TB	1.75		
	(2–10) TB	2.02		
	(1‐20) TB	1.37		
	(110) TB	1.12		
	SF TB	4.82	Growth	Pinned by S lattice
Incoherent high‐index GB		High	Growth	Pinned by dislocations
Incoherent Low‐index GB	(1–10) (010) GB	1.88	Mechanical	Mobile

**Figure 3:**
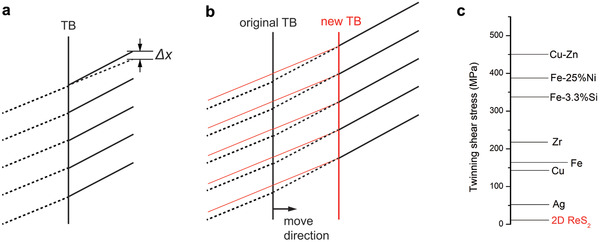
Mechanically induced TBs in 1L ReS_2_ a) Schematic of displacement (Δ*x*) of the twining nucleation under share stress. b) TB movement under shear stress. c) Comparison of twinning shear stress in 2D ReS_2_ with other metals and inorganic materials ^[^ ^38^ ^]^.

As we mentioned, the shear stress is responsible for the twining nucleation and TB movement (**Figure** **3**a,b), and normally the energy cost for twinning nucleation are much higher than TB movement.^[^ ^36^ ^]^ According to the Peierls framework for twinning,^[^ ^37^ ^]^ the critical stress for twinning can be estimated by

(1) $$\begin{array}{c} \tau \left(\mathrm{pristine}-\mathrm{twinned}\right)=\frac{\Psi \left(\mathrm{twinned}\right)-\Psi \left(\mathrm{pri}\right)}{\Delta x\left(\mathrm{twinned}\right)-\Delta x\left(\mathrm{pri}\right)} \end{array}$$

Where *τ* is the shear stress along the slip planes, Δ*x* is the share displacement of central pair of planes, Ψ is the energy of sheared configurations. The relationship of stress and energy is an integral in Peierls concepts which turns to linear during the calculation between two different orientations. Using the DFT obtained energies and lattice distortions, the critical twinning stresses in 2D ReS_2_ are estimated between 4 and 30 MPa. These values are much lower than the conventional twinning stresses for metals or inorganic bulks,^[^ ^38^ ^]^ (Figure 3c) in line with the high‐frequency twinning in 2D ReS_2_ as we have observed. The normal in‐plane dislocation slip energy barriers in 2D TMD materials are higher than 0.5 eV/dislocation cores,^[^ ^39^ ^]^ which is significantly over the dislocation slip barriers in conventional bulk metals.^[^ ^40^ ^]^ Thus the highly mobile GBs and immobile dislocations in 2D ReS_2_ can be rationalized, as we will discuss in the following.

**Figure 4:**
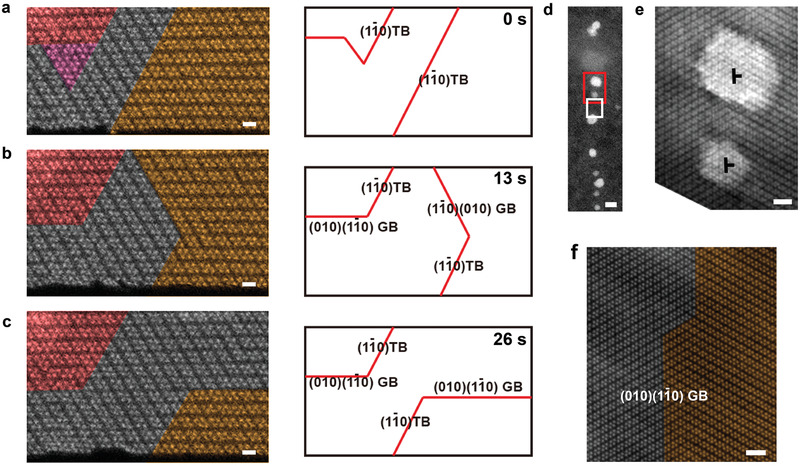
In situ STEM observations on the kinetics of GBs in 1L ReS_2_. a–c) A serial in situ STEM‐ADF atomic scale observation on the mobile GBs next to the free edge of 2D ReS_2_ during 26 s. Scale bars: 0.5 nm. Right panels highlights the mobile GBs correspondingly. d–f) The dislocation pinned incoherent low‐index GB. e,f) Magnified images for red and white rectangles, respectively. Scale bars in (d): 3.5 nm, (e,f):1 nm.

**Figure 5:**
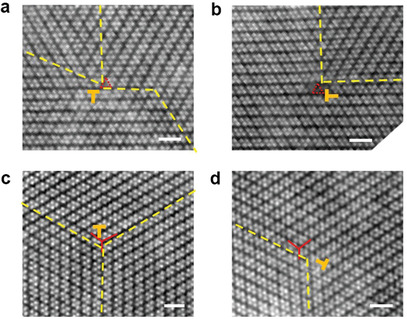
High resolution STEM‐ADF images for pinned GBs by defects in 2D ReS_2_. The immobile dislocations are marked by yellow signs. Yellow dashed lines highlight the GBs. Scale bars = 1 nm.

By using in situ STEM technique (Experimental Section; Figure S5, Supporting Information), the mobile GBs have been real‐time observed, which nucleated and moved close to the free edges of 2D ReS_2_ (**Figure** **4**a–c). During our in situ observations, the TBs and incoherent low‐index GBs ((1–10)(010) GB) can freely move under the impact of external straining (by electron beam effect on the peripheral areas). No apparent defects or dislocations were generated inside the area of interest (the observation area shown in Figure 4a–c). However, in another case (Figure 4d,e), an incoherent (1–10)(010) GB was observed to be pinned by series of dislocations (each dislocation was covered by the bright hydrocarbon residual particles) (Figure S6, Supporting Information). Therefore, the kinetics of the group of coherent or incoherent low index GBs are highly dependent on the environment close to the GBs. Due to the lattice mismatch, long incoherent GBs tend to be pinned by (partial) dislocations, while the short incoherent GBs, especially low‐index GBs are highly mobile and can actively participate in the lattice reconstructions in 2D ReS_2_ under external mechanical loading. More examples on defects (dislocation) pinning can be found in **Figure** **5**. The GBs presented are predominantly coherent TBs or low index incoherent GBs, pinned by the dislocations resulted from the CVD growth (Figure 5b,d,e) or dislocation interactions (Figure 5a,c). Same mobile GBs are also observed in the area not near the free edge (Figure S7, Supporting Information). Meanwhile, it is noticed for both mobile and pinned GBs, once they are formed, it will be difficult to remove them. However, by controlling the growth condition to obtain single crystal samples during synthesis and try to avoid the mechanical strain processing in the material, the detrimental GBs for devices could be prevented.

Disregarding the surface (edge) effect, all of the straight GBs inside the 2D ReS_2_, irrespective of the GB types, are thermodynamically stable. Kinetics of GBs will be either triggered by the reduction of local curvatures in GBs (driven by GB tensions), or by the external mechanical stresses. Furthermore, these mobile GBs can be pinned by the relatively immobile dislocations, which is opposite to the cases in traditional metals. Usually in bulk metals, the dislocation dynamics (slip) are slowed down by the relatively immobile GBs or TBs.^[^ ^41^ ^]^ Therefore, the introduction of GBs or TBs intentionally can be utilized for material strengthening. However, in 2D ReS_2_ here, the inversed sequence for twinning (or lattice reconstruction which produce the incoherent low‐index GBs) and the dislocation slip under mechanical deformation have eventually led to the abnormal defect dynamics as we observed.

**Figure 6:**
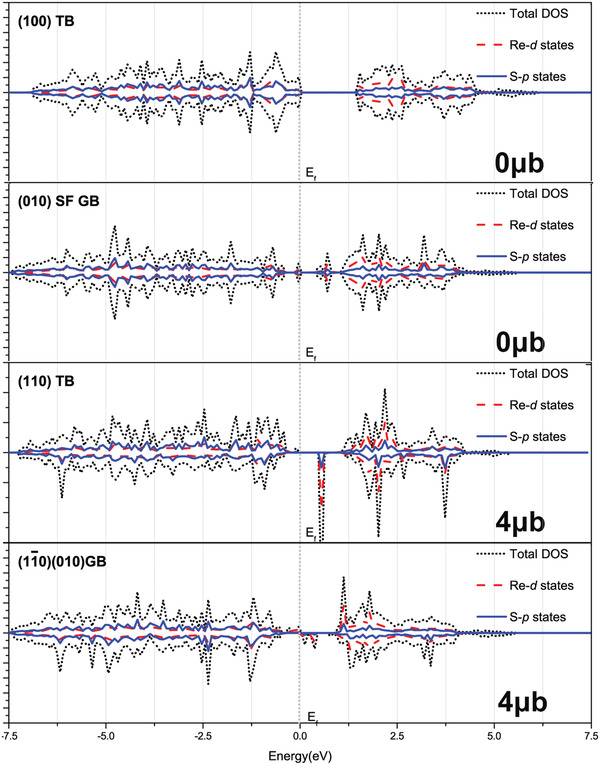
The Spin‐polarized density of states (DOS) calculated by the DFT for various GBs (complete results in Figure S8 in the Supporting Information). DOS (black dot line) and local DOS projected on 5d (red dash line) states of Re as well as 3p of S (blue line) of ReS_2_. The Fermi level is set to zero. Calculated magnetic moments are marked in lower‐right.

We have further investigated the electronic structures by spin‐polarized DFT calculations (see Experimental Section) for the coherent low‐index, coherent high‐index and incoherent‐low index GBs, respectively (**Figure** **6**; Figure S8, Supporting Information). The band structures and the magnetic moments vary widely within different types of GBs. Some GBs have very prominent deep levels, like the (110) TB. While some do not have mid‐gap states, like the (100) TB. The deep levels are the keys to the electrical transport control. While the bandgaps also vary from 1.3 to 1.8 eV. Interestingly, three kinds of TBs and the misfit incoherent low index GBs have very large magnetic moments, suggesting the opportunities in magnetic property modulations using mechanical approaches.

## 3 Conclusions

In conclusion, we have established a complete library for the GBs in monoclinic 2D ReS_2_ and rationalized the kinetics of GBs. Different from the immobile GBs in high‐symmetry 2D materials previously, the GBs in 2D ReS_2_ can be formed by either bottom‐up growth or mechanical loading, and the notable kinetics of GBs are also dependent on the GB types. In particular, the dislocations as well as S lattices can pin the GBs. In this connection, the material properties (e.g., electrical, optical, and mechanical properties) will not only be passively impacted by the GBs, in turn, the GBs in 2D ReS_2_ can be created and changed under exterior straining, thereby extending rich possibilities for strain engineering on the electrical, electronic, and magnetic properties in such 2D monoclinic materials.

## 4 Experimental Section

##### Synthesis of 2D Rhenium Disulfide (ReS_2_)

2D 1L ReS_2_ was grown by method of atmospheric CVD on a c‐face sapphire substrate with a size of 1 cm × 1 cm. The precursor for the two quartz boats were ammonium perrhenate (NH_4_ReO_4_) (Aldrich, 99.999%) and sulfur powder (Aldrich, 99.998%), with 1:50 of the weight ratio. The temperature of sulfur and substrate were precisely controlled by a two‐zone splitting tube furnace. The sulfur zone temperature rose to 200 °C, while the substrate temperature was held for 10 min at 850 °C. Argon gas was used as carrier gas to deliver the sulfur vapor to substrate during deposition process.

##### (S)TEM Specimen Preparation

Through the well‐known PMMA‐assisted technology, CVD‐grown ReS_2_ was transferred to the TEM grid. Using spin coating, a thin layer of PMMA formed on the grown sapphire substrate at 3000 rpm for 50 s. Supported by PMMA, 1L ReS_2_ was immersed in deionized water at 75 °C for two hours and fell off the substrate. The PMMA/ReS_2_ layer was then transferred to a Quantifoil TEM grid and dried at ambient temperature. In the end, the PMMA film was gently removed by acetone vapor.

##### (S)TEM Characterizations

The STEM JEM‐ARM200F with aberration correction at an acceleration voltage of 60 kV was used which equipped with a CEOS spherical (Cs) aberration corrector. The vacuum during the measurement was kept at 1.3 × 10^−7^ mbar, and the electron beam current was 13 µA. The size of the scanning probe is ≈1.5 Å. For image acquisition, the camera length on STEM was 120 mm. With a defocus of −4 nm, the acquisition time of HAADF images is 19 µs per pixel to minimize damage and obtain graphics with lower drift. Images of 512 × 512 pixels were obtained with a CL aperture of 40 µm, and the collection angle ranged from 45 to 180 mrad, to make atomic images with appropriate contrast be obtained. Wiener filtering was applied to HAADF images to reduce noise.

##### In Situ STEM on Free Edge

Exposing 2D ReS_2_ under the ultrahigh beam intensity more than 0.3 pA nm^−2^ for 15–30 min can create controlled circle area with diameter about 100 nm. After that, microscope was quickly changed into STEM mode. Due to the crack dynamics outside the exposed area of the beam, the length of these newly generated crack edges extended. Under the in‐plane mechanical loading, the shear stress caused the lattice reconstruction in 1L‐ReS*_2_*. With the in situ STEM serial capture (time interval 13–20 s), the GB dynamics under shear stress can be recorded.

##### STEM Simulation

The STEM simulation was conducted in QSTEM.^[^ ^42^ ^]^ The high voltage of simulation was 60 kV, with −4 nm of defocus. The spherical aberration was 1 µm, Cc is 1 mm, and the temperature was 300 K. Angle of detectors was set from 45 mrad to 180 mrad, meanwhile the convergence angle was 15 mrad. The TDS runs 30 rounds for each simulated image, exhibited under the brightness of 10 × 10^8^ A (cm^2^sr)^−1^.

##### Density Functional Theory Calculations

The first‐principles calculations were performed with spin‐unrestricted manner by using the Vienna ab initio Simulation Package (VASP) program package^[^ ^43^, ^44^ ^]^ within the projector augmented wave (PAW)^[^ ^45^ ^]^ to explore geometries and electronic properties of ReS_2_. The exchange‐correlation interactions are described with the generalized gradient approximation (GGA)^[^ ^46^ ^]^ in the form of the Perdew, Burke, and Ernzernhof (PBE) functional.^[^ ^47^ ^]^ This kinetic energy cut‐off of 450 eV was utilized in all calculations, and the distance of vacuum layer was set to be more than 20 Å, which was sufficiently large to avoid interlayer interactions. The electronic SCF tolerance was set to 10^−4^ eV. Fully relaxed geometries and lattice constant were obtained by optimizing all atomic positions until the Hellmann–Feynman forces were less than 0.02 eV Å^−1^. The *k*‐points samplings with a gamma‐centered Monkhorst–Pack scheme^[^ ^48^ ^]^ were 3 × 5 × 1 and 5 × 9 × 1 for structural optimizations and electronic properties, respectively .

## Conflict of Interest

The authors declare no conflict of interest.

## Supporting information

Supporting Information
