# Supplementary material for: The Mobile and Pinned Grain Boundaries in 2D Monoclinic Rhenium Disulfide
Source: Adv Sci (Weinh). 2020 Oct 12;7(22):2001742. doi: 10.1002/advs.202001742 (PMC7675180; doi:10.1002/advs.202001742)
Supplement: Supplementary file 1 — Supporting Information [file ADVS-7-2001742-s001.pdf]

© 2020 Wiley-VCH GmbH

Supporting Information

for *Adv. Sci.,* DOI: 10.1002/advs.202001742

The Mobile and Pinned Grain Boundaries in Two-Dimensional Monoclinic Rhenium Disulfide

Fangyuan Zheng[1+,], Lingli Huang[2,3+], Lok-Wing Wong[1,], Jin Han[4], Yuan Cai[5], Ning Wang[5], Qingming Deng[4], Thuc Hue Ly[2,3]*, Jiong Zhao[1,]**

Supplementary information for

The Mobile and Pinned Grain Boundaries in Two-Dimensional Monoclinic Rhenium Disulfide

Fangyuan Zheng[1+], Lingli Huang[2,3+], Lok-Wing Wong[1], Jin Han[4], Yuan Cai[5], Ning Wang[5], Qingming Deng[4]*, Thuc Hue Ly[2,3]*, Jiong Zhao[1,]*

[1] Department of Applied Physics, The Hong Kong Polytechnic University, Kowloon, Hong Kong, China.

[2] Department of Chemistry and Center of Super-Diamond & Advanced Films (COSDAF), City University of Hong Kong, Kowloon, Hong Kong, China.

[3] City University of Hong Kong Shenzhen Research Institute, Shenzhen, China.

[4] Physics department and Jiangsu Key Laboratory for Chemistry of Low-Dimensional Materials, Huaiyin Normal University, Huaian 223300, China.

[5] Department of Physics, Hong Kong University of Science and Technology, Clear water bay, Hong Kong, China.

[+] These authors contribute equally.

Email: jiong.zhao@polyu.edu.hk, qingmingdeng@gmail.com, thuchly@cityu.edu.hk,

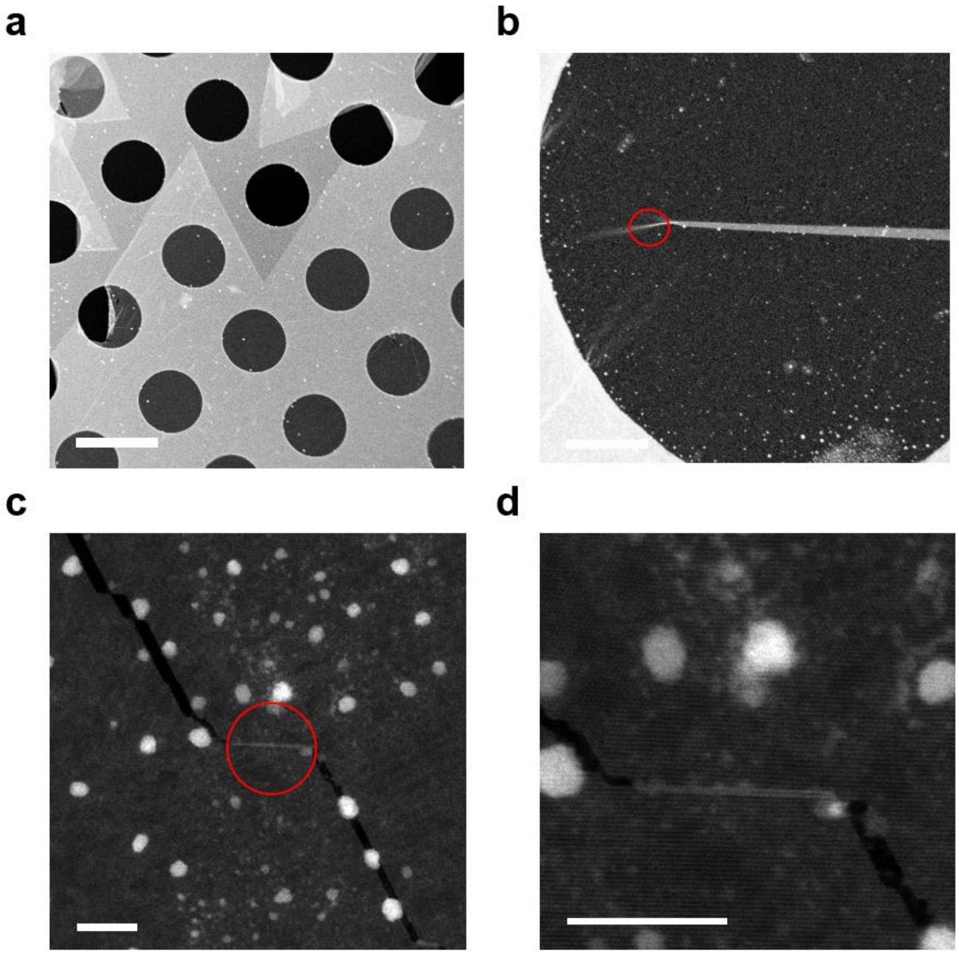

Figure S1. STEM-HAADF images of samples in low magnification and defects. a) Monolayer ReS2 flake in low magnification. b) low magnification and c) high magnification images of wrinkles, d) low magnification and e) high magnification of cracks and overlaps. Scale bar: a) 2μm; b) 200nm; c), d) 20nm.

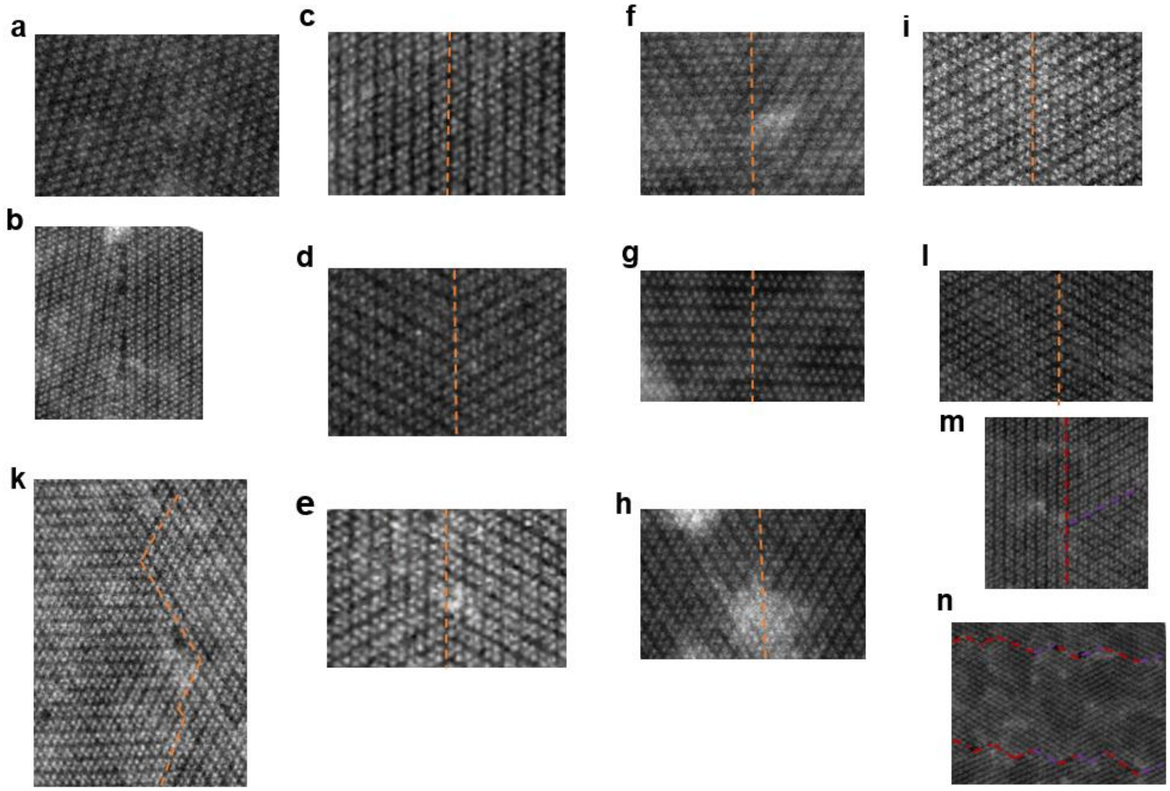

Figure S2. STEM-HAADF raw data of Figure 2.

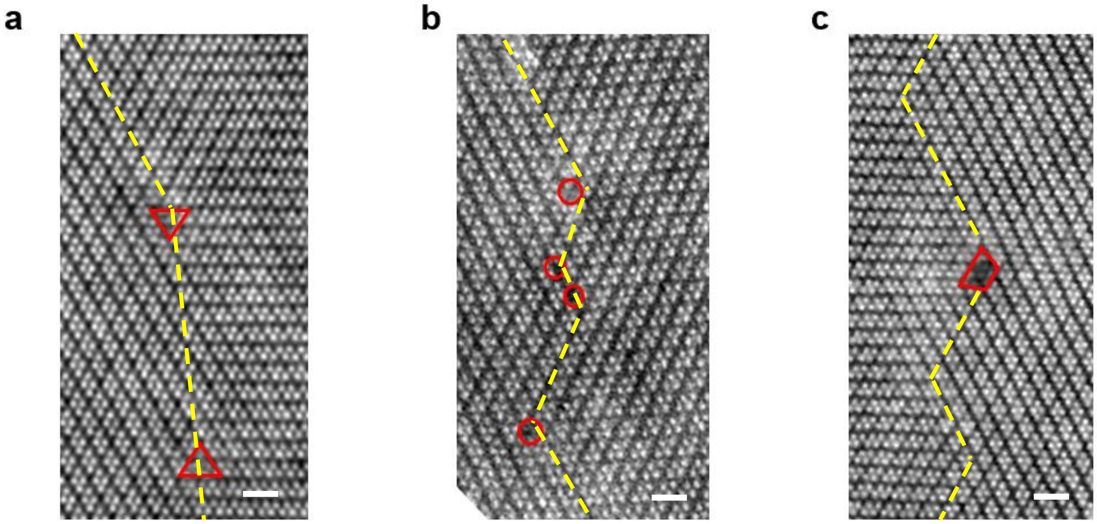

Figure S3. STEM-ADF images of zigzag segmented GBs near different dislocation cores (highlighted by red marks) in 2D 1L ReS_2. The lattice orientations are decomposed into short segments. Yellow dashed lines highlighted the GBs. Scale bars =1nm.

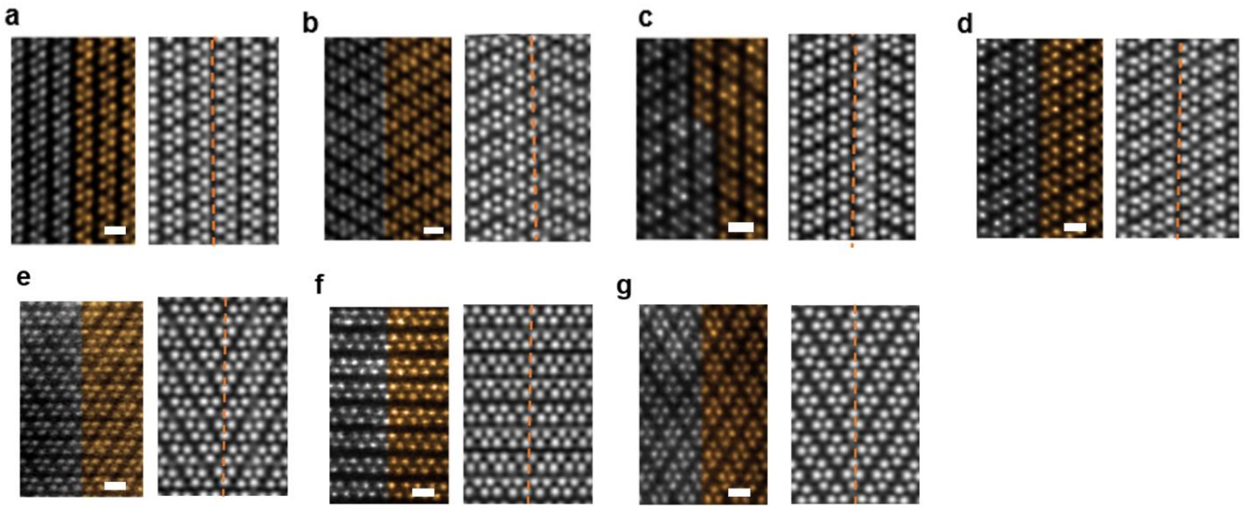

Figure S4. Comparison of STEM images and simulation of different types of GBs in ReS_2. Scale bar of STEM figures is 0.5 nm in all figures.

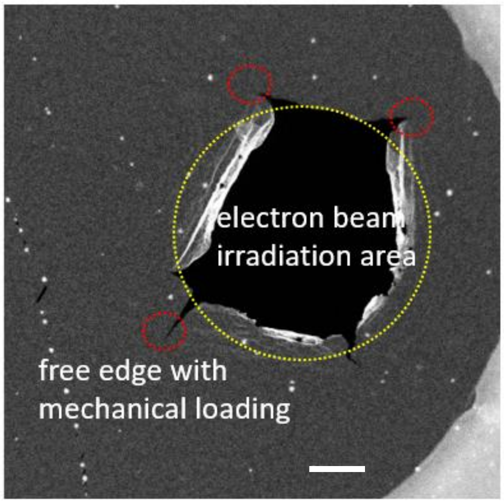

Figure S5. The *in situ* free edge method and conditions. Low magnification STEM-ADF image for 2D 1L ReS_2 after controlled beam irradiation in yellow circled area. Cracks (red circled areas) propagate from the edge of the circle to the outer area of irradiation area. Scale bar: 50 nm.

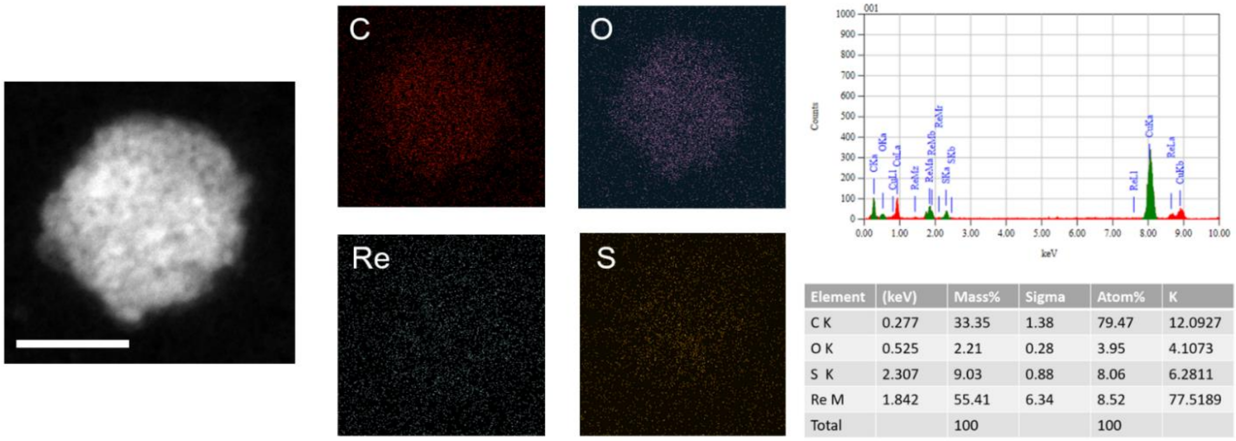

Element	(keV)	Mass%	Sigma	Atom%	K
C K	0.277	33.35	1.38	79.47	12.0927
O K	0.525	2.21	0.28	3.95	4.1073
S K	2.307	9.03	0.88	8.06	6.2811
Re M	1.842	55.41	6.34	8.52	77.5189
Total		100		100	

Figure S6. STEM-EDS mapping and element contents of contamination on ReS_2. Scare bar: 20nm.

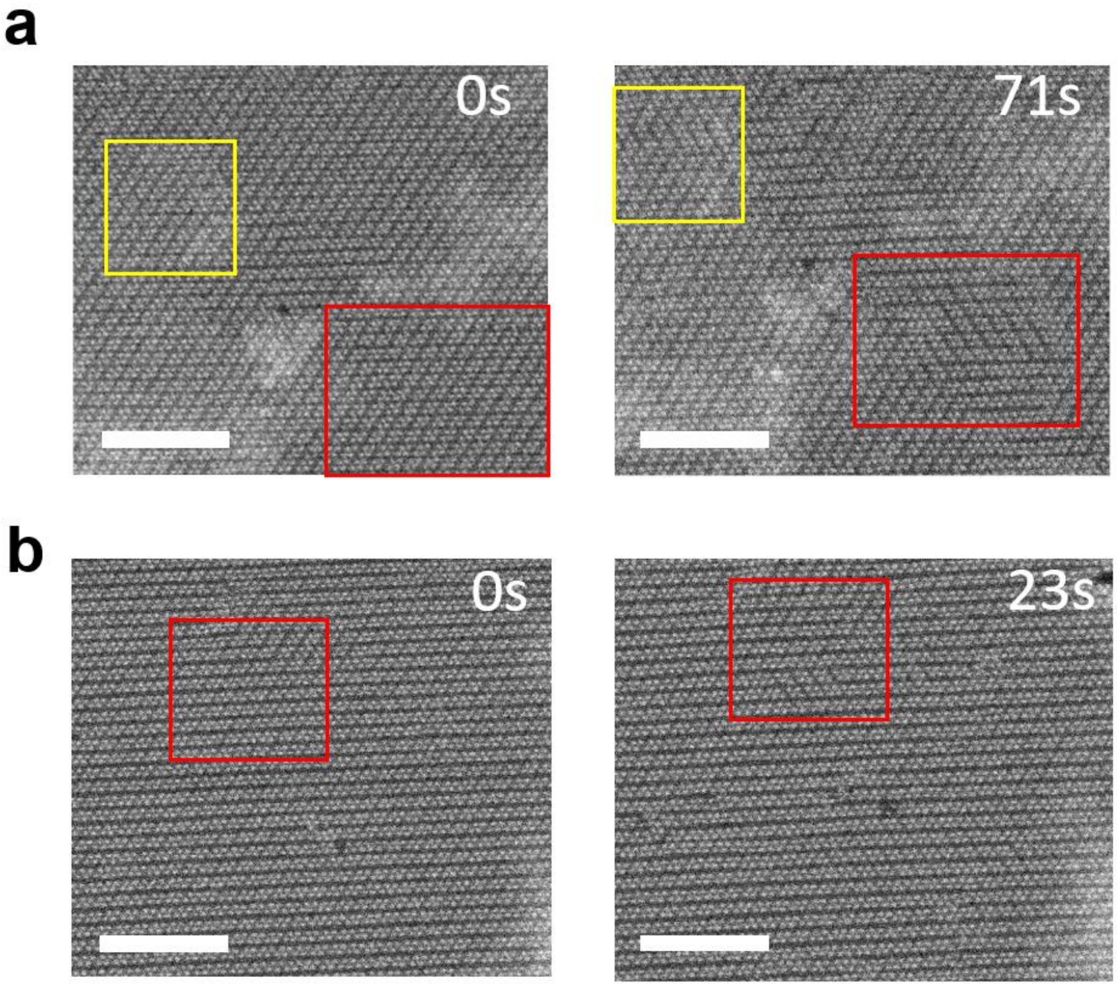

Figure S7. *in situ* STEM observations on the kinetics of GBs in 1L ReS_2. Scale bar: 5nm.

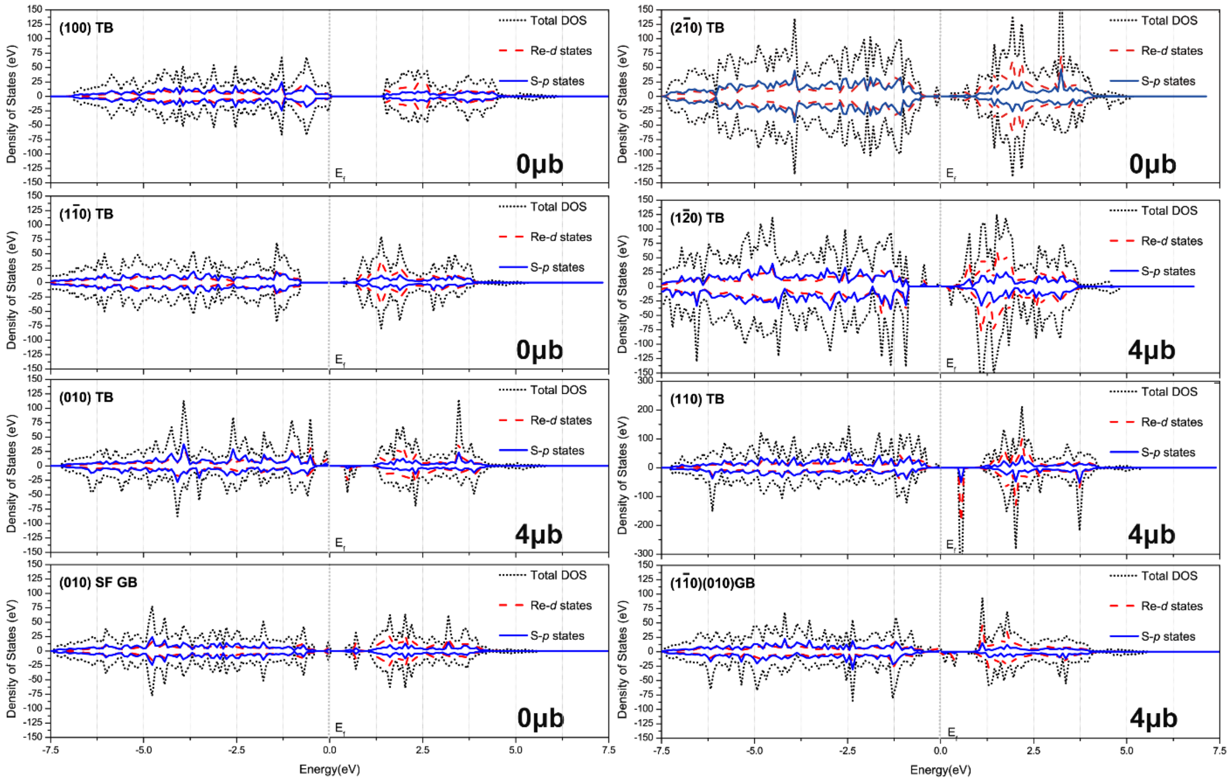

Figure S8. The Spin-polarized density of states (DOS) calculated by the DFT methods for various GBs. DOS (black dot line) and local DOS projected on 5d (red dash line) states of Re as well as 3p of S (blue line) of ReS_2. The Fermi level is set to zero. Calculated magnetic moments are marked in lower-right.
